# Critical reflections on the manuscript “Impact of the COVID-19 pandemic on quality of life and mental health in children and adolescents in Germany” published in ECAP on January 25th 2021

**DOI:** 10.1007/s00787-021-01928-x

**Published:** 2021-12-24

**Authors:** B. Platt, V. Danzer, G. Schulte-Körne

**Affiliations:** Department of Child and Adolescent Psychiatry, Psychosomatics and Psychotherapy, LMU University Hospital, Munich, Germany

We commend Ravens-Sieberer et al. [2] for embarking on a study of an important topic and collecting a large amount of clinically relevant data during difficult and uncertain times. As youth mental health researchers and clinicians we agree that the pandemic poses a threat to young people’s mental wellbeing and second the authors’ call for increased awareness and targeted mental health care. However, we believe that aspects of the study design, combined with the modest effect sizes reported and questions about the representativeness of the sample do not justify the authors’ relatively monocausal conclusions that “*children and adolescents in Germany feel significantly burdened by lockdown, social distancing and homeschooling measures*” and “experience significantly lower HRQoL and more mental health problems” (p. 7). We advise caution in the interpretation of their conclusions that the results suggest “careful balancing lockdown/homeschooling measures against the mental health risks of children” (p. 7).

## Critique of causal attribution

We observed several incidences in the manuscript where the authors imply a causal role of the pandemic and/or social distancing measures against it. For example, “To examine which children are at higher risk of being particularly **impaired by** the pandemic” (p. 4) and “Health promotion and prevention strategies need to be implemented to maintain children’s and adolescents’ mental health…and mitigate the **burden caused by **Covid-19…” (p. 1). The title refers to the “**Impact of** the COVID-19 pandemic **on** quality of life and mental health in children and adolescents in Germany”. Whilst the word “impact” is often used in public health research to refer (beyond causality) to broad associations between variables, it may imply causality to many readers of the manuscript, including members of the general public and journalists citing the study*.* Causality is best established through experimental study designs where the purported causal factor is manipulated and potentially confounding variables are kept constant [4]. However, in the case of natural disasters such as a global pandemic longitudinal data collected before, during and after a particular event are more appropriate ([1], p. 149). In this case, researchers must be careful to collect data not only on the hypothesised outcomes, but also other constructs which could affect both the independent and dependent variables and thus act as potentially confounding variables. Since the authors were unable to perform a longitudinal analysis of the 2017 sample they compared it to a different sample collected after the onset of the pandemic in 2020. Whilst this design choice is understandable and the authors acknowledge its limitations, it may still be that the observed differences in mental health simply reflect natural differences in the sample characteristics of the two samples. Furthermore, although the authors control for some crucial sociodemographic factors associated with the dependent variable(s) (e.g. age, gender), they do not control for other equally important factors which could affect the independent variable (“the pandemic”) such as other changes in population mental health between 2017 and 2020. The observed differences in mental health may, therefore, be related to non-pandemic-related changes which occurred between 2017 and 2020. In future studies, we suggest that causal inferences are reserved for longitudinal studies which follow a single sample over time and control for plausible confounding factors.

A perhaps more important aspect relating to the relatively monocausal attributions drawn is the loose definition and operationalisation of the term “pandemic” (the authors do not clearly differentiate between the causal effect of the pandemic itself and the measures taken against it such as lockdown). This is a problem because to tease apart the particular impact of anti-pandemic measures such as lockdown, the authors would need a different design. As it stands, the research literature is unclear on whether the negative effect of the social distancing outweighs the negative effect of the Covid-19 incidence on children’s mental health. It is possible that without the social distancing measures children’s mental health would have suffered more during the pandemic due to them being exposed to serious illness or death of family members, friends and educational leaders, etc. Finding a public health policy which protects children’s mental health is a complex matter. Future studies of youth mental health during the pandemic which include not only measures of social distancing but also of local Covid-19 incidence and hospitalisation levels as well as young people’s contact with those who have been infected by the virus could provide valuable insights for public health policy.

## Discussion of results

The authors paint a relatively narrow summary of the negative impact of the pandemic on the study outcomes in the Abstract and Discussion, for example, *“Two-thirds of the children and *adolescents reported being highly burdened by the COVID-19 pandemic. They experienced significantly lower HRQoL (40.2 vs. 15.3%), more mental health problems (17.8 vs. 9.9%) and higher anxiety levels (24.1 vs. 14.9%) than before the pandemic” (Abstract).

The first potentially misleading aspect of the Abstract and summaries in the Discussion is that they do not mention that a worsening of child outcomes was not seen consistently across all hypothesised outcomes. The fact that depressive symptoms did not increase during the pandemic, which contrasted with the authors’ hypotheses, is neither reported in the Abstract, nor in the Discussion. Furthermore, it is not included in Table 4, which summarises inferential statistics regarding group differences across the two time points. Finally, whilst more than half of the children in the 2020 sample (82.8%) reported fewer social contacts during the pandemic, the majority (60.7%) felt their relationships with friends had *not* been impaired and family arguments had not increased (72.4%). Whilst this was unlikely to be intentional and we of course acknowledge the suffering of those who did report impairments, these summaries could easily lead readers to misinterpret the findings. This could particularly be the case if they have not read the methods or results sections in detail. In future studies it would be important to present the summaries of findings in a balanced way so that modest effects are acknowledged as such and outcomes which have not worsened are described.

The second limitation of the discussion of the findings is that the size of the effects is not reported in the Abstract or Discussion, despite all reported effect sizes being either small or negligible. For example, the difference found between the two samples in average mental health (SDQ) scores is a small effect (*f*^2^ = 0.04; p. 6), as are the subscales of the SDQ (e.g., hyperactivity and peer problems; *f*^2^ = 0.03 and 0.05, respectively; p. 6). Some other effects are negligible (parent-reported conduct problems and self-reported anxiety; *f*^2^ = 0.01; p. 6). These small effect sizes reflect the fact that there was substantial variation in the outcome variables within both the 2017 and 2020 sample, meaning that, e.g., for general mental health, whether data were collected in 2017 or 2020 explained just 10.5% of the variance in scores. Although we understand the authors may have been restricted in the Abstract word count, some basic narrative information on the size of key effects would have been important. International scientific guidelines for example recommend that effect sizes be reported in study abstracts (e.g. JARS; https://apastyle.apa.org/jars/quant-table-1.pdf). Of note, the findings regarding the proportion of children and adolescents who experienced low HRQoL (Table 2; 15.3 vs 40.2%) or noticeable mental health problems (Table 3; 9.9 vs 17.8%) before versus during the pandemic may indeed be larger effects, but we could not find effect sizes (or test statistics) in the Results section for these comparisons (only statements in Tables 2 and 3 that the difference was significant at the *p* < 0.001 level). Similarly, the decrease in mean HRQoL from 2017 to 2020 is likely to be larger than other outcomes (Beta coefficient = − 6.51; 95% CI = − 7.28 to − 5.74), however, this is not possible to judge because the authors report neither an effect size (*f*^2^) nor a specific *p* value. Could the authors please report these values?

## Sample representativity

The authors emphasise that this is the first study of the impact of the pandemic in a *representative* sample of the German population. A representative sample is important to be sure the effects found in the study accurately depict those of the general population. However, it is unclear how representative the 2020 sample is. The authors mention that “The weighted data of the final study sample matched the sociodemographic characteristics of the German population (based on the 2018 microcensus; the individual weights ranged from 0.2 to 3.8).” (p. 3). Unfortunately, the exact sociodemographic variables compared are not reported: could the authors comment on how comprehensive the matching was? More importantly, it is unclear whether the sample is representative in terms of personal affectedness by the pandemic. Could the authors comment on how representative the 2020 sample was in terms of local infection and hospitalisation rates, deaths of family members, need for emergency daycare? Although the authors mention that German-speaking families with computer literacy and availability may have been more likely to participate, could they comment on how the recruitment, testing and reimbursement procedures may have affected the representativeness of the sample?

## Summary

We commend the authors for their study of an important topic which includes a large amount of clinically relevant data collected during difficult and uncertain times. We also agree with the authors that the pandemic poses a significant threat to the mental health of young people. However, in our view, the study design and findings do not justify the authors’ relatively monocausal conclusion that the pandemic and associated social distancing measures have had a negative impact on children’s mental health. The poorer mental health outcomes reported in the 2020 sample of this study (i) may not relate to the pandemic at all or may have stemmed from pandemic-related factors (e.g. number of hospitalisations) whose role was not investigated in the study, (ii) are relatively modest effects, and (iii) may not be representative of all children in Germany during the pandemic. Importantly, it is unclear whether mental health problems would have been even worse, had it not been for the social distancing measures adopted. Of note, in a subsequent manuscript published on October 12th 2021 Ravens-Sieberer and colleagues report longitudinal data from the same (2020) sample [3]. In our view, the methods and results in this manuscript are well-described and findings discussed in a well-balanced way. We encourage other researchers in this field to adopt longitudinal study designs where possible and to investigate not only the potential impact of social distancing on mental health but also local Covid-19 incidence and sickness and death of family and friends.

## References

[1] Jhangiani R, Chiang I-CA, Cuttler C, Leighton DC, Kwantlen Polytechnic University (2019) Research methods in psychology (4th Edn). ISBN: 9781999198107 1999198107. eBook available at: https://kpu.pressbooks.pub/psychmethods4e/. Accessed 22 June 2021

[2] Ravens-Sieberer U, Kaman A, Erhart M, Devine J, Schlack R, Otto C (2021). Impact of the COVID-19 pandemic on quality of life and mental health in children and adolescents in Germany. Eur Child Adolesc Psychiatry.

[3] Ravens-Sieberer U, Kaman A, Erhart M, Otto C, Devine J, Löffler C, Hurrelmann K, Bullinger M, Barkman C, Siegel NA, Simon AM, Wieler LH, Schlack R, Hölling H (2021). Quality of life and mental health in children and adolescents during the first year of the COVID-19 pandemic: results of a two-wave nationwide population-based study. Eur Child Adolesc Psychiatry.

[4] Woodward J (2003). Making things happen: a theory of causal explanation.

